# Seasonal Effects on the Population, Morphology and Reproductive Behavior of *Narnia femorata* (Hemiptera: Coreidae)

**DOI:** 10.3390/insects8010013

**Published:** 2017-01-17

**Authors:** Lauren A. Cirino, Christine W. Miller

**Affiliations:** Entomology & Nematology Department, University of Florida, Gainesville, FL 32611, USA; lacirino@ufl.edu

**Keywords:** plant-insect interactions, coreids, sexual selection, male-male competition, sexual dimorphism index

## Abstract

Many insects are influenced by the phenology of their host plants. In North Central Florida, *Narnia femorata* (Hemiptera: Coreidae) spends its entire life cycle living and feeding on *Opuntia mesacantha ssp. lata*. This cactus begins producing flower buds in April that lead to unripe green fruit in June that ripen into red fruit through December. Many morphological and behavioral characteristics of *N. femorata* are known to be affected by cactus phenology in a controlled laboratory setting, including the degree of sexual dimorphism and mating behavior. Our goal with this study was to determine if similar phenotypic changes of *N. femorata* occurred over time in the wild, and the extent to which these changes were concordant with phenological changes in its host plant. Further, we investigate the length of the insect mouthparts (beak) over time. Ongoing work has suggested that beak length may change across cohorts of developing insects in response to feeding deep within cactus fruit where seed and pulp depth decrease as the fruit ripens. Our results revealed a drop in cactus fruit abundance between the months of July through October 2015 as cactus fruits turned red and ripened. Simultaneously, the average body size of both males and females of *N. femorata* declined at two sampled sites. Male hind femora (a sexually-selected weapon) decreased disproportionately in size over time so that males later in the year had relatively smaller hind femora for their body size. The sex-specific patterns of morphological change led to increased sexual-size dimorphism and decreased sexual dimorphism for hind femora later in the year. Further, we found that beak length decreased across cohorts of insects as cactus fruit ripened, suggesting phenotypic plasticity in mouthpart length. Behavioral studies revealed that female readiness to mate increased as the season progressed. In sum, we found pronounced changes in the phenotypes of these insects in the field. Although this study is far from comprehensive, it provides tantalizing patterns that suggest many directions for future research.

## 1. Introduction

Plants typically change seasonally and so does the nutrition that they provide to herbivores [1,2,3,4]. As plant quality declines, herbivore mortality can increase [5] and traits vital for reproduction can also be affected [6,7,8]. Many insects temper such fluctuations in diet quality through migration [5,9], while others transition to another type of food plant [10]. However, certain insect populations do not migrate nor change their host plants across seasons. Instead, they cope with the phenological changes of their host.

Foundational work on insects including aphids [11], forest caterpillars [12], leafrollers [13], and apple maggot flies [14] have revealed the many intriguing ways that insects have adapted to seasonal changes in host plants [15,16]. Recognizing the dynamic ecological relationships between animals and their environment has provided insights into phenotypic plasticity and evolutionary adaptation [17,18,19]. Our aim for this study was to track major seasonal changes in a host plant while also recording abundance, morphological, and behavioral changes in a hemipteran, the leaf-footed cactus bug, *Narnia femorata* Stål (1962, Figure 1) [20].

*N. femorata* ranges from the southern United States in California, New Mexico, Arizona, Texas and Florida to Central America in Mexico and Costa Rica [21,22,23,24,25]. *N. femorata* was most likely introduced to Florida on nursery stock in the 1960s [22]. *N. femorata* had become fully established in Florida in the 1980s by feeding on the prickly pear cactus, *Opuntia mesacantha ssp. lata* (Small) Majure 2014 (formerly referred to as *O. humifusa*) [22,26]. *N. femorata* has since been combined with *N. pallidicornis* retaining the species name *femorata* [27].

Florida populations of *N. femorata* overwinter as adults at the base of its host plant, *O. mesacantha ssp. lata* [22,28]. Upon the arrival of spring, the overwintering adults will oviposit in rows on the spines of cactus, sticks, or pine needles that have fallen near cacti [22,29]. *N. femorata* are bivoltine in the southwest [22,30,31] and the first generation reaches maturity about two months after oviposition [22,31]. These hemipterans develop in five characteristic instars starting with a non-feeding first instar that lasts 4–7 days [31,32]. The subsequent four instars last approximately one to two weeks each [31,32]. However, the duration is highly temperature dependent [29,33]. Male *N. femorata* grapple with one another using their spiny enlarged hind legs to acquire a territory—a cactus pad with ripe or unripe fruit [34]. Females fly among cactus patches to mate, feed, and oviposit. Males will frequently mount and make genital contact with females [35]. Females that are ready to mate will open up their genital plates and allow male genitalia to penetrate [35]. Successful males will turn 180 degrees and may remain attached for up to several hours [35]. Females control whether or not a genital contact results in a mating. They can refuse to mate in a variety of ways, including simply keeping their genital plates closed, striking males with their legs, running away from males, and pushing their abdominal tip down against the cactus surface [35]. They can also dislodge males once they have started to mate [29].

In North Central Florida, *N. femorata* feeds on the fruit of *O. mesacantha ssp. lata* [22,26,36]. It feeds through a long tube-like beak that helps it gain access to the nutrients deep within their host plants’ tissue and functions through the use of an osmotic pump [22,28]. *O. mesacantha ssp. lata* flowers in the late spring and fruit ripens throughout the summer and into the fall. Fruit availability begins to decline after its peak in June [35]. One reason for the decline is that *N. femorata* is not the only cactus fruit consumer. Deer, tortoises, birds, coyote, and rodents all compete for this valuable food [29,37,38,39,40]. Variation in the abundance of these vertebrate consumers over space should lead to differences in the number of cactus fruit available for *N. femorata*. For this reason, cactus fruit is expected to vary not only seasonally, but also spatially.

Developmental diet is now known to have a large effect on the phenotypes of adults in many Coreidae, including effects on size, shape, internal anatomy, attractiveness to conspecifics, and mating behaviors [8,35,41,42,43,44]. A developmental diet with cactus fruit is very important for juvenile *N. femorata* [34]. Juveniles can feed on the cladodes (pads) of *Opuntia* spp., but without any fruit during the final stages of development they mature into tiny adults, males have greatly reduced weapon size, and sexual dimorphism is diminished [8,32,45]. Pilot data has suggested that beak (mouthpart) length appears to be phenotypically plastic, changing with the presence and phenology of cactus fruit [46]. *N. femorata* is proving to be a valuable laboratory study organism for investigating questions in the field of sexual selection, yet few details of the natural history and seasonal changes of wild populations have been provided until now [31].

Based on previous and ongoing work in this coreid and others, we predicted that seasonal and spatial variation in their food should be associated with a host of phenotypic changes in the wild. We predicted that a dramatic decline in fruit numbers in the field would also be associated with a dramatic decline in the body size of these insects. We also predicted that mouthpart length would decrease in the late summer and fall because as fruit ripens, the outer tissue layer of the fruit becomes thinner and allows the insect to reach the pulp and seeds at a shallower point [47]. Furthermore, because the availability of high quality food should be linked to population growth and size [48], we expected to find more adults following the months when cactus fruit was plentiful.

We established two distinct sites for our study that differed strikingly in land use. At these sites, we noted changes in cactus fruit phenology and fruit abundance over time. We also visited these sites monthly, counting and measuring insects, and bringing insects back to the lab to test their readiness to mate. We see this study as a first step towards understanding how the differences in these plants over time and space may be related to phenotypic differences in wild populations of *N. femorata* and the success of these populations.

## 2. Materials and Methods

### 2.1. Study Sites

**Figure 2:**
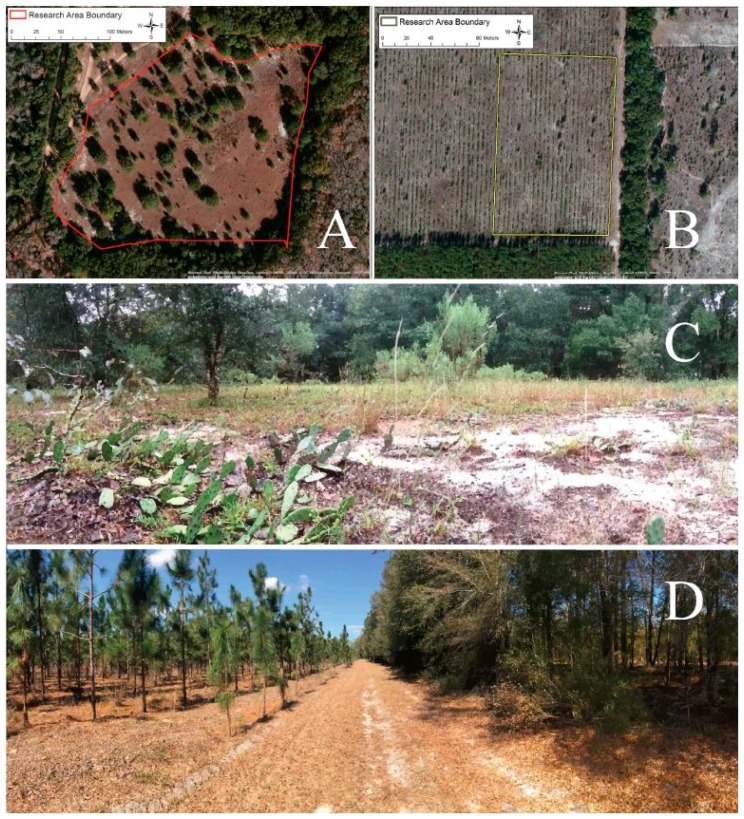
Aerial and understory photos of both Protected (**A** & **C**) and Agricultural Site (**B** & **D**). Lines drawn on the aerial photographs illustrate the survey areas at each site.Photo credit **A** and **B**, Google Earth. Photo credit **C** and **D**, Pablo Allen.

All of the survey work was completed in North Central Florida in 2014–2015 [22]. Our primary site included a 41,338 square meter survey area and nearby cactus patches at the University of Florida Ordway-Swisher Biological Field Station in Melrose, Florida (Figure 2A). Fire management is one of the ecologically important practices carried out at this site which leads to routine burning and scarring of cactus plants. A combination of dormant and growing season prescribed burns maintain the longleaf pine-wiregrass community on the Station. We refer to this area as the “Protected Site.” *A priori* our goal was simply to measure temporal changes in insect phenotypes. However, we discovered a new field site during the work, and we seized the opportunity to add it for the last four months of the study to provide a spatial dimension to the work. This site is referred to as the “Agricultural Site.” The Agricultural Site included a 14,385 square meter survey area and nearby cactus patches at Future Farmers of America (FFA) agricultural plot in Live Oak, Florida (Figure 2B). This area is a pine plantation; longleaf pine trees are grown and harvested at this site every 15–18 years. Prickly pear cactus, *O. mesacantha ssp. lata*, are also unintentionally grown. While both sites have sandy soil, the two sites are strikingly different in plant community composition and structure. The Protected Site is comprised of a diverse mix of oaks, palmettos, pines, grasses, and herbaceous plants (Figure 2C); whereas, the Agricultural Site has reduced diversity, permitting growth of only a few species beyond longleaf pine trees and prickly pear cactus (Figure 2D). Importantly, herbivore abundance and community composition are likely quite different at the two sites. The Protected Site boasts abundant wildlife, including tortoise, deer, birds, and rodents, all important consumers of cactus fruit. Although the soil nutrient content of the sites is unknown, neither site has been artificially fertilized in the past ten years [49].

### 2.2. Cactus Patch Sampling Study

Our objective for this sampling work was to estimate changes in the phenology and number of cactus fruit of *O. mesacantha ssp. lata* over time. Twenty cactus patches outside of the insect sampling area (see below) were marked in each site; these twenty locations were chosen based on the presence of *N. femorata* at the onset of the study. A cactus patch was defined as a group of cactus pads within 25 cm of each other [50]. We revisited these cactus patches in the third week of each month. We counted the number of fruit found on the patches of *O. mesacantha ssp. lata* and roughly categorized each fruit as 1) flower blooms and unripe green fruit or 2) ripe red fruit. These cactus patches were located nearby the plots where the insect sampling study was completed.

### 2.3. Insect Sampling Study

In a separate sampling study, our objective was to estimate the changes in the abundance and track the morphological changes of wild *N. femorata* adults and nymphs over four months (July through October) during the period of time when the insects are most active. Our sampling area at the Protected Site was 41,338 square meters (Figure 2A), and our sampling area at the Agricultural Site was 14,385 square meters (Figure 2B). We sampled these larger areas to be able to achieve a more representative estimate of changes in *N. femorata* abundance than would be possible from counting insects on just the twenty patches used in the patch sampling work. We haphazardly surveyed *O. mesacantha ssp. lata* cactus pads for *N. femorata* for 3.5 to 4 hours at each site. The overall total number of nymphs and adults found at each site were recorded each month. Adults were collected by hand, placed in collection vials, sexed by determining whether or not they possessed a genital slit (indicative of females).Body size (i.e. pronotum width or PW) and weapon size (i.e. hind femora width or HFW) were estimated using Mitutoyo digital calipers (maximum accuracy 0.01 mm) as insects were held immobilized. Pronotum width has been shown to be an excellent proxy for body size for *N. femorata* in laboratory studies [34,35]. Hind femora width is simple to measure, sexually dimorphic, and representative of overall hind femora area [45]. This work was completed in a day during the second week of each month. We marked insects before releasing them to ensure we did not measure the same insect during multiple sampling periods [see also above]. Only five of the 353 sampled insects were re-sighted during subsequent surveys. Of these five, three were recaptured one month later, one was recaptured two months later, and one was recaptured four months later.

### 2.4. Readiness to Mate and Beak Length

Female mate choice and male-male competition are affected by resource availability and quality in *N. femorata* in laboratory settings [34,35]. Females prefer the odor of males that develop on cactus fruit and are even more likely to choose males when red ripe fruit is present [34,35]. Males are more likely to engage in competition for cactus territory with red fruit than those territories with unripe green fruit or no fruit at all [51]. Our aim for this part of the study was to determine the extent to which female readiness to mate and male mating attempts changed over time in insects originating from the wild, not including predicted changes due to age and mating status [52]. To minimize potential effects of age and to control for mating status, we collected fifth instar nymphs and brought them back to the lab to complete development on red ripe fruit until reproductive maturity. Adults become reproductively mature between 10 and 21 days from adult eclosion [53], thus nymphs spent approximately two to four weeks in the lab in standardized conditions before testing.

Insect collection was completed monthly during the months of July through October for the readiness to mate and beak length studies (see below), when the insects were most abundant. We use “cohort” to describe the juvenile insects collected at each time point (four cohorts total). We standardized nymph care across all months; we kept the fifth-instar nymphs individually in an environment composed of a deli cup, soil, a cactus pad with high quality red ripe fruit, and they were stored in a greenhouse (14 hours light: 10 hours dark across the four cohorts). Thus, our care regime should dilute seasonal changes in these insects, and most conclusions regarding changes in female and male readiness to mate should be conservative. If readiness to mate is largely determined in the fifth instar or early adulthood by factors that differ over time in the field but not in the lab, then we would not see a change in readiness to mate over time using our focal cohorts. In other words, a lack of change could be due to an actual lack of change for wild populations or a result of the time spent in the lab.

Once individuals became sexually mature adults, between 17 and 27 days [53], we randomly paired age-matched adult males and females. Pairs were age matched within six days of each other based on date of eclosion. Tests of readiness to mate were performed by pairing a single male and a single female in a cup with a cactus pad and red fruit while a single observer documented behaviors. Single pairs of females and males on cacti are common at the Protected Site, though not as common at the Agricultural Site where the population density is high [29].

These trials were carried out for 3 hours in an 80 degree Fahrenheit room that was fully lit with two sets of florescent lights: ceiling and low hanging. We determined female readiness to mate by whether or not the female allowed copulation upon male mounting. Thus, for this analysis, we only included those pairs where males mounted, where females had the opportunity to show a preference. A male mounting was recorded when the male placed at least two of his legs and at least 50% of his body on top of the female. We determined male readiness to mate by whether or not the male attempted to mount the female, and all pairs were included in this analysis.

Next, we examined beak length, providing the first study tracking beak length over time in wild populations of *N. femorata*. We predicted that beak (mouthpart) length would be shorter as fruit ripened in the field. We used the same insects that had been collected for the readiness to mate trials to estimate the extent to which beak length varies temporally. Detecting changes in beak length was contingent on the developmental effect persisting through a final few days of rearing on a standardized diet in the lab. We measured body size and beak length (BL) on frozen *N. femorata* by first photographing the frozen specimens using a digital camera (Canon EOS 50D, Canon, Tokyo, Japan) attached to a dissecting microscope (Leica M165C, Leica Biosystems, Nussloch, Germany). Next we took body measurements using ImageJ software [54] (v1.42d, NIH, Bethesda, MD, USA). Prior to each set of pictures taken, we photographed a scale bar to ensure accuracy of the measurements.

### 2.5. Statistical Analyses

We examined body size and weapon size in our wild population of insects using a generalized linear model with site and month as categorical explanatory variables. We ran separate models for males and females given the known sexual size dimorphism in this species [45]. We first examined the effects of site (Agricultural Site, Protected Site), month (July, August, September, and October), and site × month on body size and weapon size separately. We next constructed a model to test for changes in the size of weapon size relative to body size (i.e., body shape differences). We built the initial models with body size as a continuous covariate, site and month as categorical factors, and all two-way interactions of site, month, and PW. Two-way interactions where *p* > 0.10 were removed from models using a sequential elimination procedure [55]. We calculated the sexual dimorphism indexes (SDI) of the body size and the weapon size of insects measured in the wild and the SDI of beak lengths from the separate group of insects measured in the lab (see above). To calculate the SDI, we divided the average female trait size by the average male trait size per month per site and subtracted 1 [48].

To examine male and female readiness to mate over the four successive cohorts and from the two sites, we used a generalized linear model with a binary response variable and logit-link function. Our response variable for males was mating attempt (yes/no), and our response variable for females was readiness to mate (yes/no) following a male mating attempt (predictor variable).

We next examined body size and beak length (BL) from the cohorts of insects brought in the lab for the readiness to mate trials. We ran separate generalized linear models for males and females. We first examined the effects of site, month, and site by month on body size and BL separately. We next constructed a model to test for changes in the scaling relationship between body size and BL across cohorts and sites. We built the initial models with month and body size as continuous covariates, site as a categorical factor, and all two-way interactions of site, month, and body size. Again, the sequential elimination procedure was used for model reduction. All analyses were completed using IBM^®^ SPSS^®^ ver. 22 (IBM, Armonk, NY, USA).

## 3. Results

### 3.1. Fruit Number and Phenology of O. Mesacantha Ssp. Lata

**Figure 3:**
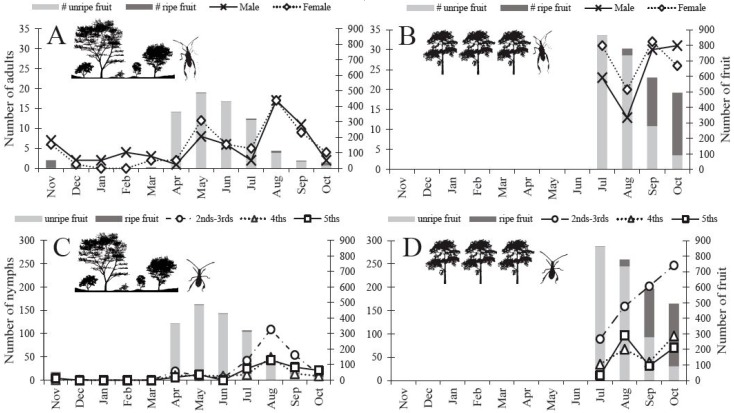
*N. femorata* abundance (per sampling location) and cactus fruit phenology (per 20 cactus patches) from the insect sampling study 2014–2015 for both the Protected Site and the Agricultural Site. Both cactus blooms and green fruit were classified as ”unripe fruit” (light gray bar), “ripe fruit” (dark gray bar) includes only red cactus fruit. (**A**–**B**) *N. femorata* abundance for adults (**C**–**D**) and nymphs in 2014–2015. (**A**) Adults in the Protected and (**B**) Agricultural sites and (**C**) nymphs in the Protected and (**D**) Agricultural sites with fruit abundance matched to the corresponding month. Insects and cactus fruit phenology were not sampled November–June at the Agricultural Site.

Flower blooms started to form on *O. mesacantha ssp. lata* in the warmer months of April and May and were replaced by green unripe fruit in June [29]. Green unripe fruit began to turn red and ripen in July, and gradually red ripe fruit became more numerous than green unripe fruit (Figure 3). We found a small number of red ripe fruit throughout the entire year (Figure 3A,C). The difference in fruit availability was striking across the seasons and across the two sites. The Agricultural Site had more overall fruit available per 20 patches of sampled cacti in July (~ 169%), August (~ 588%), September (~ 1106%), and October (~ 1235%) than the Protected Site (Figure 3).

### 3.2. Abundance of N. femorata

Adults were present throughout the entire year, yet we detected low numbers during the winter months of December through March (Figure 3A). By May, the numbers began to increase and spike in August at the Protected Site (Figure 3A). The Agricultural Site carried a higher number of adults throughout all months of the summer and into the fall relative to the Protected Site (Figure 3B). Unlike the adults, nymphs were visually absent in the winter months (Dec–Mar) in the Protected Site (Figure 3C). Although we did not formally sample the Agricultural Site in the winter months (because this site was incorporated after the winter), we observed nymphs in the following year’s fall/winter during visits conducted in November and February. Nymphs began to appear in April in the Protected Site (Figure 3C). We detected a peak in nymph abundance twice in the Protected Site: once in April and again in August (Figure 3C). Interestingly, we did not detect peaks in abundance at the Agricultural Site, rather nymph numbers appeared to increase through the fall months (Figure 3D). We found more adult *N. femorata* at the Agricultural Site versus the Protected Site in July (~ 671%), September (~ 210%), and October (~ 850%), but fewer adults in August (~ −3%). We also found more nymphs of all developmental stages in the Agricultural Site versus the Protected Site for July (~ 71%), August (~ 61%), September (~ 183%), and October (~ 860%).

### 3.3. Size and Sexual Dimorphism of N. Femorata

**Figure 4:**
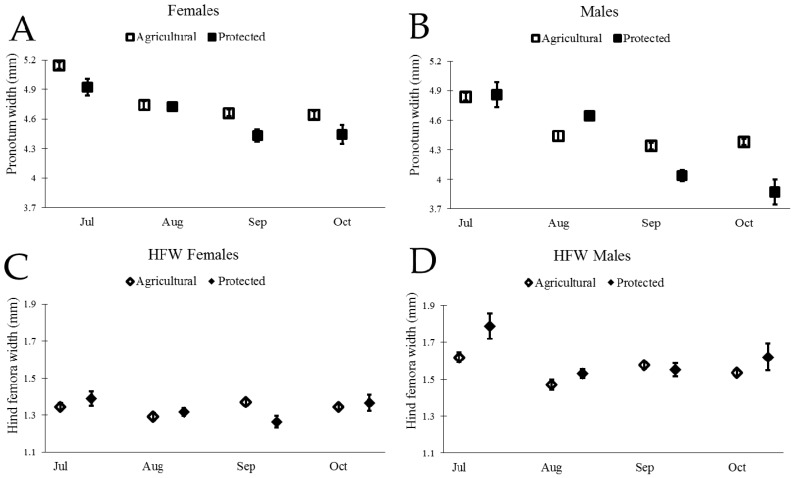
Body size and weapon size for both male and female *N. femorata* for both Agricultural and Protected sites. (**A**) and (**B**) Mean changes (±SE) in wild caught *N. femorata* of pronotum width (body size) from GLM analysis (no covariate) for A) females and B) males over time. Graphs (**C**) and (**D**) show estimated marginal means (EMM) (±SE) in wild caught *N. femorata* for hind femora width (weapon size) at the mean body size (**C**) 4.77 mm for females and (**D**) 4.47 mm for males over time.

Wild adult insects measured in the field were larger in July than in September and October, as reflected in both measurements of body size and weapon size (Figure 4 and Table 1). We found that the extent to which size decreased over time differed across the sites for most traits (significant effect of site × month; Table 1).

We next examined the scaling relationship between weapon size and body size for both males and females from the two sites, following similar procedures used by Miller et al. (2016) [45]. Our initial model included the main effects of body size, site, month, and all two-way interactions. Interactions of body size with site or month would indicate that the scaling slope of body size and weapon size changed across sites or across months. We did not find evidence of a change in scaling slope (PW > 0.10 for interactions between PW and month for both males and females), thus we sequentially removed the interactions of body size with site and body size with month and proceeded to test for a change in intercept due to site, month, or both factors. We indeed found evidence of changes in the intercept of weapon size with body size, illustrating in particular that males in July had proportionately larger weapon size for a given body size (Figure 4 and Table 2).

**Figure 5:**
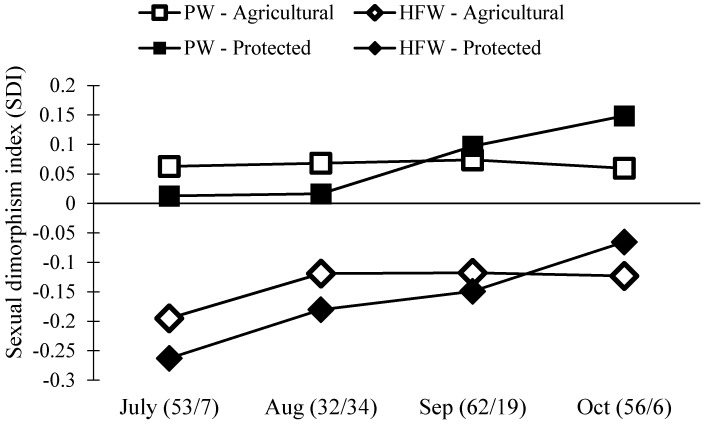
Population level sexual dimorphism index (SDI) of two traits, pronotum width (PW) and hind femora width (HFW), at both the Protected and Agricultural Sites. If the number is below zero, males have the larger trait. If the number is above zero, females have the larger trait. The numbers in the parentheses are the sample size for each month (Agricultural/Protected).

Separate size analyses of males and females do not directly compare males and females across sites and months. Thus, we next estimated the level of sexual dimorphism in *N. femorata* using a well-known metric, the sexual dimorphism index [56]. We found a surprising change in sexual dimorphism across our two field sites and over time (Figure 5). In particular, the level of dimorphism in the weapon size decreased over the season. We also found that females became disproportionately larger than males at the Protected Site over time.

### 3.4. Beak Length in N. Femorata

**Figure 6:**
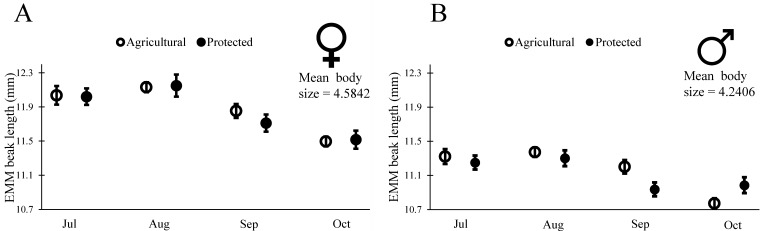
Estimated marginal means (±SE) of beak length in wild caught 5th instars reared on red ripe fruit until a reproductively mature adult in the laboratory. Values are generated from generalized linear models run separately for each sex and controlled for the mean body size for that sex (Table 4).

Insects used in the readiness to mate trials (above) had been originally collected as fifth-instar nymphs from the field. They molted to adults within a week of being brought to the lab, and at that point had their final beak size for life (as is characteristic for most insects). We used this group of insects to examine changes in beak length across our four cohorts of insects. To determine if phenotypic changes in these insects were representative of changes in wild populations, we also examined body size for these insects. As we saw with the wild-measured adults (Table 1 and Figure 4), insects collected in July had larger body size (PW) than those in the subsequent cohorts (males: Wald χ^2^ = 18.331, df = 1, *p* < 0.001; females: Wald χ^2^ = 13.90, df = 1, *p* = 0.003), though the change appeared reduced for these partially lab-reared insects. In July, female body size was on average 4.85 mm at the Agricultural Site and 4.74 mm at the Protected Site, and male body size was on average 4.57 mm at the Agricultural Site and 4.45 mm at the Protected Site. By October, female body size was on average 4.54 mm at the Agricultural Site and 4.51 mm at the Protected Site, and male body size was on average 4.23 mm at the Agricultural site and 4.24 mm at the Protected Site. As with body size, we found that beak length decreased with subsequent cohorts (Table 3), as would be expected for a trait that scales with body size. To determine if beak length decreased relative to body size we proceeded to examine the scaling relationship between BL and body size for males and females from the two sites. Our initial model included the main effects of body size, site, month, and all two-way interactions on BL. Interactions of body size with site or month would indicate that the scaling slope of body size and BL changed across sites or across months. We did not find evidence of a change in scaling slope (PW > 0.10 for interactions between PW and month for both males and females), thus we sequentially removed the interactions of body size with site and body size with month and proceeded to test for a change in intercept due to site, month, or both factors. We indeed found evidence of changes in the intercept of BL with body size, revealing that both males and females had relatively shorter mouthparts in later months. In other words, not only did insects have shorter beaks in the later months, but the beaks were shorter relative to their body size (Table 4, Figure 6).

### 3.5. Female and Male Readiness to Mate in N. Femorata 

Ninety-seven pairs of insects were paired for readiness to mate trials over the months of July through October. Seventy-four percent of males (72 of 95 males) attempted to mate with females during our observation window (26% did not). The males that did not attempt mating were scattered across sites and across months, though with a peak in August (males that did not attempt mating: 2 of 20 in July (10%), 12 of 30 in August (40%), 4 of 21 in September (19%), 7 of 26 in October (27%). All males from the Agricultural Site attempted mating in July, and all males from the Protected Site attempted mating in August, thus we could not conduct a generalized linear model with both site and month as explanatory variables (i.e. there were empty cells). We proceeded with separate analyses of effects of site as a categorical variable and month as a continuous variable and found no statistically-significant evidence that male readiness to mate (mating attempts) was affected by site or month (site: Wald χ^2^ = 2.145, df = 1, *p* = 0.143, month: Wald χ^2^ = 0.246, df = 1, *p* = 0.620).

Seventy-two males attempted to mate, and only nine females refused. All females accepted mating attempts in the August and September cohorts from the Protected Site and in October for the cohort from the Agricultural Site. The lack of variation in responses across these months and sites (empty cells) prevented analysis of month and site simultaneously in a generalized linear model. Thus, we analyzed the effect of site as a categorical variable and month as a continuous variable with separate generalized linear models. We found that female readiness to mate increased across the cohorts: 13 of 18 in July (72%), 16 of 18 in August (89%), 16 of 17 in September (94%), and 18 of 19 in October (95%) (Wald χ^2^ = 3.860, df = 1, *p* = 0.049). We found no evidence that female readiness to mate was affected by site (Wald χ^2^ = 0.307, df = 1, *p* = 0.580).

## 4. Discussion

*Narnia femorata* abundance and phenotypic characteristics were remarkably dynamic in the wild across two sites and over time. The extent to which these changes are directly related to host plant phenology, temperature, social environment, or other factors cannot be concluded from this single study. However, many results mirror findings from the laboratory [4,8,33,34,35,36,43,45,57]. Together, the existing work makes a powerful case that host plant phenology can alter growth and development in these insects, and that these phenomena extend to real-world settings. We found expected trends over time in the phenology and abundance of cactus fruit [35], and we also found pronounced spatial differences in cactus fruit abundance.

### 4.1. Size and Sexual Dimorphism

We found a remarkable decrease in body size and the size of the sexually-selected weapon (hind femora) of male *N. femorata* over time (Figure 4B,D and Table 1). Laboratory studies have shown that insects that develop without cactus fruit during their final instars are stunted in growth [45]. The data presented here suggest that the loss of cactus fruit over time in the field has similar consequences for wild insects. The loss of cactus fruit between July and September was particularly pronounced at the Protected Site where 91% of fruit was removed from our focal cacti. In comparison, the Agricultural Site had only 45% of fruit removed from the focal cacti. The decline in male body size and weapon size was greater at the Protected Site than at the Agricultural Site (Figure 4B,D), as would be predicted from a valuable resource being so drastically depleted. The body size and weapon size of adult insects do not change over time (they are set for life after the final molt), so the patterns seen here likely reflect the influx of new, stunted juveniles eclosing to adulthood in our population. Size-dependent mortality could also be a factor changing the average trait values in our population.

Male *N. femorata* appear to be particularly sensitive to poor nutrition [45]. Here, both males and females captured later in the season were smaller than those measured earlier, yet males were affected to a greater extent than females (Figure 4). In July at the Protected Site, males were large with large sexually-selected weapons (Figure 4B,D). Sexual size dimorphism was minor at this time, with females marginally bigger than males (Figure 5). Yet, males in July had much larger hind femora than females (Figure 5). By October at the Protected Site, females were much larger than males and the dimorphism in the sexually-selected weapon shrank (Figure 5). Interestingly, the weapon size of males decreased relative to male body size so that there was a shift in the allometric scaling relationship (Figure 4D). Males in October were not only tiny, but they had especially tiny hind femora relative to males in July. The size of female hind femora relative to body size also changed across the months, but the shifts were subtle (Figure 4C).

Not all effects on body size and weapon size witnessed here were predicted by laboratory results from work on this species. For example, previous work predicted a decline in the dimorphism of weaponry with cactus fruit removal, but not a decrease in the body size of males relative to the body size of females (Figure 5) [45]. The decrease in the size of the male weapon relative to body size was also more pronounced here than predicted from laboratory results [45]. Temperature, density, or other environmental factors may add to or interact with effects of nutrition on these populations of insects and warrant further study. Finally, thepresence of some red ripe fruit, particularly at the Agricultural Site, should be a boon for insects fortunate enough to develop on this high-quality resource [8,35,36,45,57]. It may be that these well-nourished individuals existed, but that they were outnumbered by those stranded for part of development without cactus fruit at all.

The degree of sexual dimorphism may have numerous effects on sexual selection dynamics and evolution in natural populations, yet rapid spatial and temporal changes in sexual dimorphism have been given insufficient attention [45,58,59,60,61]. In one of very few examples, sexual size dimorphism in the blowfly was shown to decrease as temperature increased, which may allow for increased fecundity and relaxation of sexual selection [62]. More studies of environmental effects on sexual dimorphism and sexual selection are needed, especially those that embrace the complex ecological interactions that many species face in the wild [63].

### 4.2. Beak Length

Insects in the later cohorts were not only smaller with shorter beaks, but they also had relatively short beaks for their body size (Figure 6, Table 3 and Table 4). Intriguingly, the changes in beak length relative to body size appear to correspond to changes in *O. mesacantha ssp. lata* phenology. Since *Opuntia* spp. fruit does not increase sugar content until the end of their development [47], *N. femorata* may need longer beaks to reach a more sugar or nutrient-rich portion of fruit early in the year. The fruit’s outer layer of tissue also decreases in thickness upon maturity, which may make shorter beaks more efficient for *N. femorata* to access the nutrients within *Opuntia* fruit [47]. The rapid changes in beak length here suggest that phenotypic plasticity, not evolution, is responsible for the changes seen. Such plasticity could be an adaptation that allows the insects to make the most of their changing food.

This is one of the first times that a temporal shift in a plant resource has been documented to influence changes in insect mouthparts within one year in the wild [64]. Mouthparts appear particularly malleable over evolutionary time [65], and increasing data suggest that phenotypic plasticity in mouthpart size and shape may be common as well [66,67,68]. Phenotypically plastic changes in mouthparts have been documented in other animals in the wild such as wood frogs and spotless starlings [66,67,68]. This study may provide a mechanism by which *N. femorata* can capitalize on different *Opuntia* spp. fruits across their range and over time as the fruits mature.

### 4.3. Readiness to Mate

Most males (74%) attempted matings, and most females (88%) were ready to mate when males attempted to mate. We found an increase in female readiness to mate for later cohorts of insects, a linear trend from 72% to 95% from July to October. This increase in readiness to mate occurred in spite of the fact that males in the later cohorts were smaller with smaller weapons, and while previous studies have shown that females are less likely to mate with small males [35]. These results suggest that females may adjust their preferences depending upon the pool of males available or that female readiness to mate is so heightened in later cohorts as winter nears that mate preferences diminish [69]. Similarly, in a fiddler crab, females change their preferences for males over time, potentially due to their offspring’s developmental temperature requirements [70,71]. Yet, in general, female mate sampling may decrease temporally because it reduces the energetic costs of finding a mate during a period of time when males are scarce [72].

### 4.4. Abundance and Natural History

Other foundational work on *N. femorata* in New Mexico has shown that their abundance is responsive to changes in the reproductive effort (e.g., meristems allocated for reproduction versus growth) in the tree cholla cactus, *Cylindropuntia imbricata* (Haw.) F. M. Knuth 1935 (= *O. imbricata* (Haw.) D. C. 1828), with an increased or decreased population size depending on whether or not more fruit is available [30,41]. The results we report here show a similar pattern. The Protected Site had fewer adults overall than the Agricultural Site, perhaps due to a low number of cactus fruit. As fruit numbers declined, adults became almost absent at the Protected Site (Figure 3A). Larger *O. mesacantha ssp. lata* fruit numbers at the Agricultural Site also appeared to support a larger and increasing population of nymphs; whereas, the decreasing number of cactus fruit at the Protected Site may be responsible for declining nymph numbers after August (Figure 3C,D). Incidentally, we noticed a large number of the invasive *Cactoblastis cactorum* move into our field sites recently, largely decimating cactus pads and leading to a great reduction in fruit. We have yet to know the full consequences of *C. cactorum* on this community [73,74,75,76], and future work is needed.

## 5. Conclusions

This study provides insight into the intimate relationship between *N. femorata* and its cactus host in Florida. We hope this work will serve as a catalyst for future studies of this species in the wild, including a more in-depth analysis of the ecological interplay between *N. femorata* and its host plant. Further, the results reported here suggest that the shape and size of other insects may change in complex ways with host plant phenology, patterns that have rarely been investigated and deserve greater attention.

## Figures and Tables

**Figure 1:**
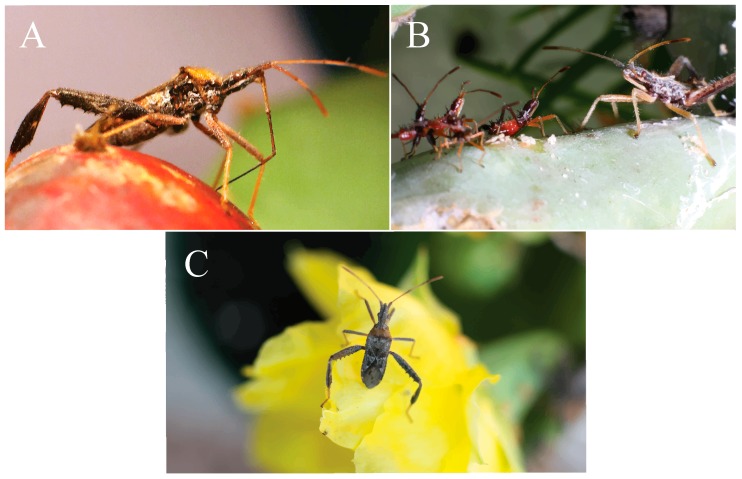
*N. femorata* in different life stages. (**A**) Adult *N. femorata* with its beak probing a red ripe fruit. (**B**) Nymph *N. femorata* in the third instar (left) and fifth instar (right). (**C**) Adult *N. femorata* on a cactus bloom. All photos credited to Christine W. Miller.

**Table 1:** Results of generalized linear analyses to examine changes in body size (pronotum width; PW) and weapon size (hind femur width; HFW) across sites and months.

Source	Male PW			Male HFW			Female PW			Female HFW		
	χ^2^	df	*p*	χ^2^	df	*p*	χ^2^	df	*p*	χ^2^	df	*p*
Site	7.868	1	0.005	0.458	1	0.499	15.814	1	<0.001	2.964	1	0.085
Month	123.178	3	<0.001	67.216	3	<0.001	81.086	3	<0.001	27.591	3	<0.001
Site × Month	40.652	3	<0.001	25.322	3	<0.001	6.213	3	0.102	15.238	3	0.002

**Table 2:** Results of generalized linear analyses to examine changes in weapon size (hind femur width; HFW) relative to changes in body size (PW), i.e., changes in insect body size.

Source	Male HFW			Female HFW		
	χ^2^	df	*p*	χ^2^	df	*p*
PW	56.663	1	<0.001	25.239	1	<0.001
Site	6.801	1	0.009	0.033	1	0.856
Month	28.132	3	<0.001	7.950	3	0.047
Site × Month	7.408	3	0.060	12.828	3	0.005

**Table 3:** Results of generalized linear analyses to examine changes in beak length (BL) across sites and months.

Source	Female BL			Male BL		
	χ^2^	df	*p*	χ^2^	df	*p*
Site	0.945	1	0.331	0.002	1	0.964
Month	13.900	3	0.003	37.527	3	<0.001
Site × Month	2.708	3	0.439	7.911	3	0.048

**Table 4:** Results of generalized linear analyses to examine changes in beak length (BL) relative to changes in body size (PW), i.e., changes in insect body shape.

Source	Female BL			Male BL		
	χ^2^	df	*p*	χ^2^	df	*P*
PW	88.376	1	<0.001	201.434	1	<0.001
Site	0.190	1	0.663	0.745	1	0.388
Month	54.593	3	<0.001	42.213	3	<0.001
Site × Month	1.041	3	0.791	9.162	3	0.027
